# Sedation with etomidate-fentanyl versus propofol-fentanyl in colonoscopies: A prospective randomized study

**Published:** 2015

**Authors:** Nadia Banihashem, Ebrahim Alijanpour, Majid Basirat, Javad Shokri Shirvany, Mehrdad Kashifard, Hasan Taheri, Shahriyar Savadkohi, Vahid Hosseini, Seyed Sedigheh Solimanian

**Affiliations:** 1 Department of Anesthesiology, Babol University of Medical Sciences, Babol, Iran.; 2 Department of Internal Medicine, Babol University of Medical Sciences, Babol, Iran.; 3 Inflammatory Diseases of Upper GI Tract Research Center , Mazandaran University of Medical Sciences, Sari, Iran.; 4 Babol University of Medical Sciences, Babol, Iran.

**Keywords:** Propofol, Etomidate, Colonoscopy, Sedation

## Abstract

**Background:**

The combination of propofol-fentanyl for sedation during colonoscopy is characterized by high prevalence of side effects. Etomidate-fentanyl provides fewer hemodynamic and respiratory complications. The aim of our study was to compare the safety and efficacy of propofol-fentanyl and etomidate-fentanyl for conscious sedation in elective colonoscopy.

**Methods:**

This double-blind clinical trial was conducted on 90 patients aged between 18- 55 years old who were candidates for elective colonoscopy. Patients were randomized to receive sedation with fentanyl plus propofol or etomidate. Two minutes after injecting 1 micro/kg of fentanyl, the patients received 0.5mg/kg propofol by infusion (25 µ/kg/min) or 0.1 mg/kg etmoidate (15 µ/kg/min). Pulse rate, mean arterial blood pressure, respiratory rate, and saturation of peripheral oxygen (SPO_2_) were monitored. In addition, the patient and colonoscopist satisfaction, the recovery time, sedation and pain score in both groups were assessed.

**Results:**

Sedation score in propofol group was higher. Pain score as well as the physician and patient satisfaction showed no significant difference between the two study groups. Hemodynamic changes and arterial saturation were the same in both groups. The duration of recovery was 1.27±0.82 minutes in the etomidate group; versus 2.57±2.46 minutes in the propofol group (P=0.001). Recovery time in the etmoid group was 2.68±3.14 minutes and in the propofol group was 5.53±4.67 minutes (p=0.001).

**Conclusion:**

The combination of fentanyl and etomidate provides an acceptable alternative to sedation with fentanyl and propofol with the advantage of significantly faster recovery time, in the outpatient setting.

Colonoscopy is one of the most commonly performed outpatient method for the diagnosis and treatment of colorectal disorders (1-4).  It is an invasive and short-lasting procedure that causes pain, restlessness, anxiety and vasovagal reactions. Sedation and analgesia are often required to successfully perform the procedure. (2-3). A combination of midazolam and meperidine provides adequate sedation during colonoscopy (1-4). Despite satisfactory comfort for most patients, it is not ideal for most patients undergoing colonoscopy (1-3). The duration of the effects of these drugs is usually longer than the time required for the procedure, and this may result in delayed recovery with a prolonged discharge time. Indeed, this combination increases the likelihood of respiratory depression (1-3). 

Over the past years, propofol was widely used for sedation because of its pharmacological characteristics and rapid recovery profile (5). The combination of propofol- fentanyl was used in many medical centers for sedation of patients under colonoscopy. However, its hemodynamic and respiratory side-effects are high and there is no reversal agent for propofol (6-7). Etomidate has rapid onset and short recovery time and minimal cardiovascular event (6). It seems that etomidate is a good sedative drug for patients under colonoscopy if combined with fentanyl. Unlike propofol-fentanyl, the use of etomidate-fentanyl for sedation in colonoscopy has not been evaluated yet. The aim of the present study was to compare the patient comfort, recovery and the safety profiles between the propofol-fentanyl and etomidate-fentanyl in patients under elective colonoscopy. 

## Methods

This randomized double-blind study was conducted after institutional ethics committee approval and written informed consent. The IRCT code of the study is: IRCT201212195381N4. One-hundred patients (aged 18–55 years) who were scheduled for elective colonoscopy were included in this study. Exclusion criteria were refusal to provide informed consent, allergy to propofol, pregnancy, obesity, neurological or psychological disorders as well as drug abuse and those with a history of colon surgery. Randomization was done using a computer-generated random number table and patients were separated into two groups: propofol group and etomidate group 

All patients received fentanyl 1 micro/kg intravenously for analgesia. In the propofol group, a bolus dose of propofol 0.5 mg/kg was given over 30s, followed by a continuous infusion at 25µg/kg/min. In the etomidate group, a bolus dose of etomidate 0.1 mg/kg was given over 30s, followed by a continuous infusion at 5 µg /kg/min. The gastroenterologists, nurses and patients were all blinded to treatment randomization. Because of the obvious differences in the appearance of the study drugs, the anesthesiologist was not blind to the study drugs. Oxygen at 4 L/min by nasal cannula was administered throughout the procedure. 

The extent of sedation was assessed by the use of Ramsay sedation Score (6). Ramsay sedation scale (1=awake, 2= drowsy, 3= arousable to command, 4= arousable to tactile stimulation, 5= not arousable) was measured and recorded at 1, 2, 5, 10, 15, 20 min after starting the drug infusion. Pain was defined as a feeling of physical hurt. The quality of analgesia was assessed using the visual analog scale (0 no pain, 10 maximum pain). The patient satisfaction (1=unacceptable, 2=very uncomfortable, 3=slightly comfortable, 4= no discomfort) was assessed 24h after the procedure by telephone interview (6). All colonoscopies were performed by one skilled colonoscopist. Endoscopist satisfaction was evaluated immediately after colonoscopy, which was assessed by a four point scale (1=poor, 2=fair, 3=good and 4=excellent). Satisfaction was graded by evaluating the ease of insertion and the patient’s lack of motion (6). 

Heart rate, mean arterial blood pressure, respiratory rate and peripheral oxygen saturation were monitored in the endoscopy and recovery room. A change in blood pressure or heart rate by 20% above or below the baseline was considered significant. 

When oxygen saturation was below 92% for more than 10 seconds or when apnea for more than 20 seconds, infusion of drug was discontinued and the jaw thrust maneuver and ventilation with mask were started. Nausea and vomiting were examined by observation and asking the patient. An independent anesthesiologist was responsible for recording the sedation score, cardiorespiratory and procedural data. 

Duration of colonoscopy (time interval between “start of colonoscopy” and “colonoscope removal) and caecal intubation time (time to reach caecum) recorded. Recovery of sedation and hospital discharge was assessed using the Modified Aldrete Scoring System and the Discharge score respectively (8, 9). The patients with a modified aldert score of 8 or more were admitted to the phase II recovery unit. The patients with a discharge score of 9 or more were discharged. The data were analyzed using the SPSS 17 software program. T-test was used for quantitative factors with normal distribution and the Mann-Whitney test was used for quantitative factors with abnormal distribution.  For qualitative factors, the Chi-square test was used. In all cases, a p-value of *less than *0.05 was considered to be statically significant. Unilateral Kolmogorov–Smirnov test was used to determine the normality of the distribution of samples. 

## Results

Four patients in etomidate group were excluded from the study, because of poor bowel preparation (three patients) and perforation of colon (one patient). Therefore, 90 patients completed the study, with 47 in the propofol group and 43 in the etomidate group. Twenty three (48.9%) patients in propofol group and 23 (53.5%) patients in etomidate group were males (P=0.679). The mean age in propofol group was 36.6±11.4 years old and in etomidate group was 36.6±9.7 years old (P=0.187). 

**Fig1:**
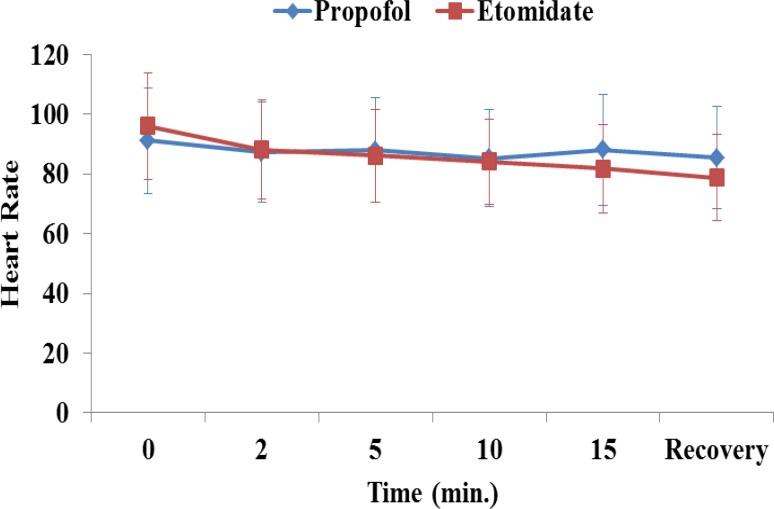
Heart rate in patients receiving etomidate or propofol

**Fig 2:**
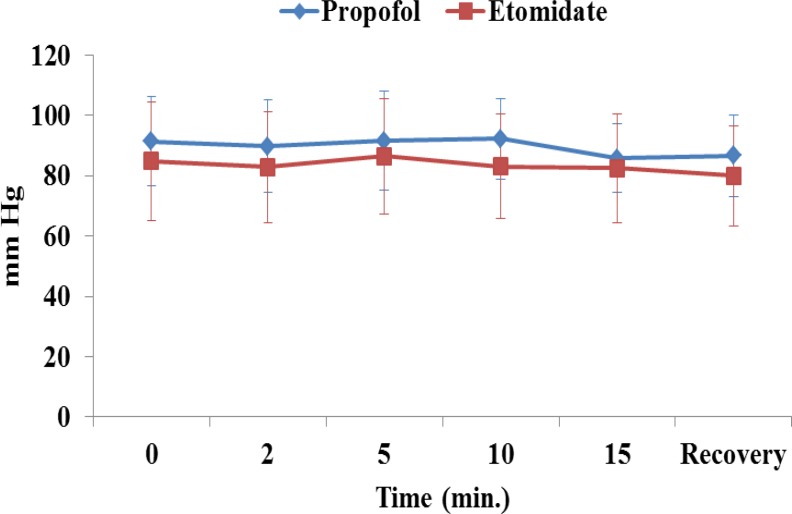
Mean blood pressure in patients receiving etomidate or propofol

**Fig 3:**
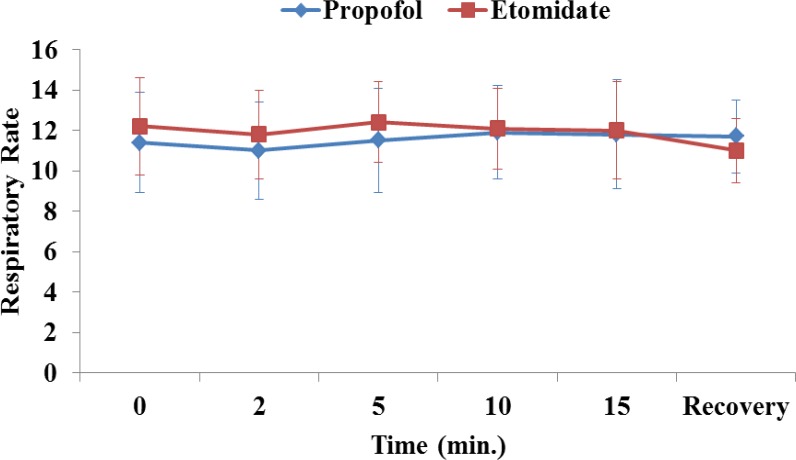
Respiratory rate in patients receiving etomidate or propofol.

**Fig 4:**
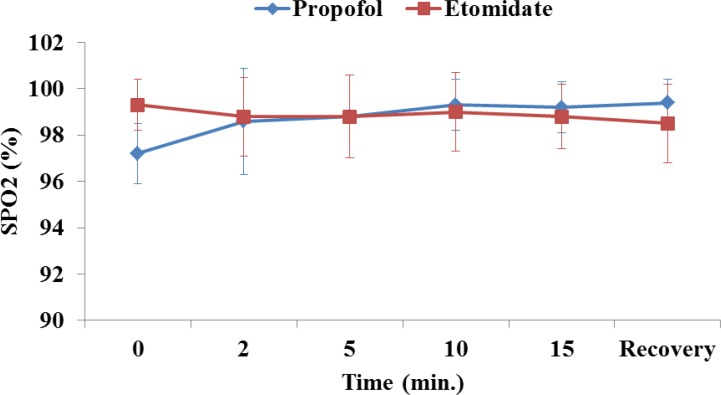
Oxygen saturation in patients receiving etomidate or propofol

The mean arterial pressure, heart rate, respiratory rate and arterial saturation remained stable during the procedure and were comparable between the groups (fig 1-4). In the propofol group, two patients have oxygen desaturation and apnea during colonoscopy (propofol group), which was corrected with stimulation and jaw thrust maneuver. 

The patients in the propofol group had higher mean sedation scores during the procedure (table 1). The maximum pain score recorded for each patient did not differ significantly between groups; all prepared to undergo the procedure again using the same form of sedation. The overall satisfaction with sedation was high. The mean comfort level, as estimated by patients, was not different for the study groups (table 2). 

The gastroenterologist satisfaction was not different in the two groups (table 2). Colonoscopy duration and cecal intubation time were summarized in table 3. Recovery and hospital discharge time were significantly shorter in the etomidate group (table 3). Four (9.3%) patients in the propofol group and ten (21.3%) patients in the etomidate group had nausea and vomiting (P=0.151). No severe complications were recorded during the colonoscopy and recovery.

**Table 1:** Sedation score in two groups

**Pvalue**	**Etomidate (n=43)**	**Propofol (n=47)**	**Time(min)**
0.002	2(1-3 )[1.58]	2(1-4)[2.34]	1
0.002	1(1-3)[1.6]	2(1-4)[2.36]	2
0.001	1(1-2)[1.37]	1(1-4)[1.94]	5
0.045	1(1-2)[1.27]	1(1-2)[1.48]	10
0.767	1(1-2)[1.32]	1(1-2)[1.37]	15
0.374	1(1-2)[1.27]	1(1-2)[1.11]	Recovery

Median (Min-Max), [Mean]

**Table 2:** Visual analog scale, patient satisfaction and colonoscopist satisfaction in two groups

**Pvalue**	**E** **tomidate**	**Propofol**	
0.85	2(0-10)[2/33]	2(0-8)[2.28]	Visual analog scale
0.22	4(3-4)[3.86]	4(2-4)[3.74]	Patient satisfaction
0.98	4(1-4)[3.44]	4(1-4)[3.45]	Colonoscopist satisfaction

Median (Min-Max), [mean]

**Table 3:** Times to ceacum intubation, colonoscopy, recovery and discharge

	**Propofol**	**Etomidate**	**Pvalue**
Ceacum intubation time(min)	5.03±1.91	6.13±4.35	0.133
Colonoscopy time(min)	9.91±2.17	11.43±4.85	0.064
recovery time(min)	2.57±2.46	1.27±0.82	0.001
Discharge time(min)	5.53±4.67	2.68±3.14	0.001

## Discussion

Our study shows that continuous infusion of propofol or etomiadate for colonoscopy can provide adequate safety. The principal results of this investigation is that the patients in the propofol group had higher sedation score. The gastroenterologist and patient satisfaction score during the procedure were comparable between the two groups. The trend of hemodynamic and respiratory variables was similar in the two groups.Recovery and time to discharge were shorter in etomidate group. 

In our study, the degree of pain and comfort level experienced during colonoscopy was not statistically different for the patients, but Ramsey sedation score was higher in propofol group. In contrast to the present study, in another study Ramsey sedation score was similar between the two groups during the study period (6). In an earlier study, Akcboy et al. showed that the patients undergoing conscious sedation for colonoscopy with remifentanil had better sedation score, analgesia and patient satisfaction compared with midazolam and propofol. They concluded that if analgesia was adequate, sedation was not required in patients during colonoscopy (10). Other studies found similar results (11, 12). In our study, the patient and colonoscopist satisfaction scores were similar in both groups. In some studies, the method of sedation had no effect on the satisfaction of the physician or patient (6, 13, 14). It seems that colonoscopist’s skill, base line pain and verbal anxiety score play an important role in patient and physician satisfaction.

 Toklu et al. found that after sedation with etomidate or propofol for colonoscopy, average recovery time was shorter in etomidate group (6). Moerman et al. found that after sedation with propofol or etomidate for cardiovertion, recovery time was shorter in the propofol group. They used etomidate or propofol with no opioids (11). In our study, recovery time and time to discharge time was longer in propofol group. Probably, higher sedation score was an important factor. 

 Benzodiazepines, narcotics and propofol in different combinations are administered to provide sedation in patients undergoing colonoscopy. According to the previous study, the most common complications in gastrointestinal endoscopy are cardiorespiratory adverse events such as hypoxemia, hypoventilation, apnea, dysrhythmias, hypotension and vasovagal episodes (14). In present study, the heart rate, blood pressure, oxygen saturation and respiratory rate were comparable in both groups and no adverse events were seen during the colonoscopies of either group. The achieved sedation in the two groups of study was at a moderate level. Lower respiratory depression and hemodynamic instability in the present study compared with other studies might be attributed to modest sedation, continues infusion and careful titration of drugs (6, 7- 10, 15). Indeed, pre-oxygenation with intranasal oxygen providing to all patients in the present study may be the key factor of the difference. The mean of procedure duration was similar in patients of both groups, but the colonoscopy duration and cecal intubation time was shorter than the other studies (6, 16). This can be explained by better cooperation of the patients and skillful gastroenterologist with 12 years’ experience. This study may be subjected to one limitation, amnesia and the adverse effects of drug may affect the response to question and confound the results. However, these adverse effects may involve both groups and the confounding effects may be minimal. 

In conclusion, the combination of fentanyl and etomidate provides an acceptable alternative to sedation with fentanyl and propofol with the advantage of significantly faster recovery time, which are of relevance in the outpatient setting.

## Notes

**Funding:** This project was supported by the Vice-Chancellery for Research of Babol University of Medical Sciences.
